# The Inverse Association of Sarcopenia and Protein-Source Food and Vegetable Intakes in the Korean Elderly: The Korean Frailty and Aging Cohort Study

**DOI:** 10.3390/nu14071375

**Published:** 2022-03-25

**Authors:** Seon-Joo Park, Junghyun Park, Chang Won Won, Hae-Jeung Lee

**Affiliations:** 1 Department of Food and Nutrition, College of BioNano Technology, Gachon University, Seongnam 13120, Korea; chris0825@hanmail.net; 2 Institute for Aging and Clinical Nutrition Research, Gachon University, Seongnam 13120, Korea; iwbstill@naver.com; 3 Department of Family Medicine, College of Medicine, Kyung Hee University, Seoul 02447, Korea; 4 Department of Health Sciences and Technology, GAIHST, Gachon University, Incheon 21999, Korea

**Keywords:** sarcopenia, elderly, Korean, food group, nutrients

## Abstract

The aging population contributes to increasing economic and social burden worldwide. Sarcopenia, an age-related degenerative disease and progressive disorder, is characterized by a reduction in skeletal muscle mass and function. This study aims to assess the association between dietary factors and sarcopenia in the Korean elderly using nationwide data. A total of 801 subjects aged 70–84 years were included in this analysis. Subjects were divided into two groups: sarcopenic and nonsarcopenic groups according to the sarcopenia criteria established by the Asian Working Group for Sarcopenia. Nutrient and food intakes were assessed using a 24-h recall method. Logistic regression analysis was used to assess the association between sarcopenia and food group and nutrient intakes. In the multivariable models, the meat/fish/egg/legume food group, vegetable group, and total food intake were inversely associated with the prevalence of sarcopenia. The high intakes of energy, carbohydrate, protein, fiber, zinc, carotene, and vitamin B_6_ were associated with the lower prevalence of sarcopenia. Therefore, consuming sufficient nutrients through various protein source foods and vegetables will help prevent sarcopenia in the Korean elderly.

## 1. Introduction

Aging is a multifactorial and complex process, and the population is aging worldwide. According to a World Health Organization report, there should be at least 2.1 billion of the elderly population in 2050 [1]. The aging population phenomenon is increasing the social and economic burden worldwide. In particular, the anticipated degree of old age dependency ratio in 2050 in South Korea is considerably high (0.77) compared with that of the United States (0.37) and that of the United Kingdom (0.47) [2]. Sarcopenia is a degenerative skeletal muscle disorder characterized by loss of skeletal muscle mass and function, resulting in falls and loss of mobility [3,4]. Sarcopenia has been implicated in the pathogenesis of neuromuscular disorders, and condition of chronic diseases. [5,6]. After the age of 60, the muscle mass reduction accelerates to about 3% per year compared to the age of 20 [7]. The major risk factor for sarcopenia is the aging process, which is related to an altered hormonal metabolism, inflammation, decreased α-motor neurons, and redox imbalance, etc. [8]. In addition, the pathogenesis of sarcopenia is also known to be related to other aggravating factors such as gender, lifestyle, and other pathological conditions [4]. Accumulating evidence suggests that a sedentary lifestyle, bed rest periods, an inadequate dietary intake of energy and protein, malabsorption of nutrients, cachexia, gastrointestinal disorders, and chronic diseases such as diabetes and obesity may promote loss of skeletal muscle mass [9].

The Korean elderly tend to have insufficient protein and calcium intake [10]. Inadequate intake of nutrients such as protein, vitamin D, calcium, and vitamin C was associated with muscle loss in elderly Korean men [11]. Recently, fruits and vegetables have been attracting attention as having a positive effect on sarcopenia [12]. However, as far as we know, very few studies have been conducted to analyze sarcopenia and dietary factors in the Korean elderly using nationwide data [11,13]. Therefore, it is necessary to propose a dietary guideline for the prevention of sarcopenia by identifying which foods or nutrient intakes are related to sarcopenia in the Korean elderly.

This study aims to assess the associations of food group intake and nutritional status with the prevalence of sarcopenia in the Korean elderly using nationwide data and to provide fundamental data to establish dietary guidelines for the prevention of sarcopenia. 

## 2. Materials and Methods

### 2.1. Participants

The Korean Frailty and Aging Cohort Study (KFACS) is a nationwide multicenter longitudinal cohort study that started in 2016. The detailed study design and process for the KFACS have been previously published [14]. Participants aged 70–84 years were recruited from sex- and age-stratified communities in urban and rural areas across South Korea. Among the 1559 participants enrolled in the KFACS in 2016, those who did not participate in the nutritional survey (*n* = 557) and those who did not have muscle mass data measured by dual-energy x-ray absorptiometry (DEXA) (*n* = 201) were excluded from the study. In total, 801 participants were included in the final analysis. The protocol of the KFACS was approved by the institutional review board (IRB) of the Clinical Research Ethics Committee of the Kyung Hee University Medical Center (IRB number: 2015-12-103), and written informed consent was obtained from all participants before they participated in the study.

### 2.2. Definition of Sarcopenia

The Asian Working Group for Sarcopenia (AWGS) established the diagnostic criteria for sarcopenia in the Asian population using muscle mass, muscle strength, and physical performance as the criteria [15]. Skeletal mass was assessed using DEXA (GE Healthcare Lunar, Madison, WI, USA; Hologic DXA Systems, Hologic, Inc., Bedford, MA, USA), appendicular lean mass was calculated as the sum of the lean mass measurements from both the arms and legs (kg), and appendicular skeletal muscle mass (ASM) was calculated by dividing the appendicular lean mass by the height squared (kg/m^2^). The cut-offs of <7.0 kg/m² for men and <5.4 kg/m² for women were used as the criteria for a low skeletal muscle mass when skeletal mass was measured using DEXA. Muscle strength was determined by the handgrip strengths of both hands, each repeated twice, using a digital grip strength dynamometer (TTK-5401; Takei Ltd., Tokyo, Japan). The mean of the four grip strength measurements was used. Cut-offs <26 kg for men and <18 kg for women were used to define a low muscle strength. Gait speed based on over 4 m with acceleration and deceleration phases of 1.5 m each was measured using an automatic timer (Gaitspeedmeter, Dynamicphysiology, Daejeon, Korea). Low physical performance was defined as a gait speed ≤0.8 m/s [16]. In this study, sarcopenia was diagnosed satisfying both a decreased muscle mass (<7.0 kg/m² for men and <5.4 kg/m² for women) and low muscle strength (<26 kg for men and <18 kg for women) according to the AWGS 2014 criteria [15]. Additionally, patients with low physical performance (gait speed ≤ 0.8 m/s) were diagnosed with severe sarcopenia. However, only few subjects (*n* = 10) were diagnosed with severe sarcopenia in this study, therefore the data are not shown. 

### 2.3. Dietary Assessment

We used the released individual food and nutrient intake data in our study. The nutritional survey and calculation methods of nutrient intakes have been reported in detail [14,17]. To explain briefly, the dietary assessment was conducted using the 24-h recall method by trained interviewers. Food intake was estimated using measuring cups and spoons, ruler, and real-size-picture of bowls and plates developed by the National Institutes of Health (NIH) and the Korea Disease Control and Prevention Agency (KDCA) and using the 24-h recall dietary assessment system of the NIH and KDCA.

Nutrient intakes (energy, carbohydrate, protein, fat, fiber, calcium, phosphorus, iron, sodium, potassium, vitamin A, retinol, carotenes, vitamin B_1_, vitamin B_2_, niacin, vitamin C, zinc, vitamin B_6_, vitamin B_12_, folate, vitamin D, vitamin E, vitamin K, and cholesterol) were calculated using food composition database based on the National Rural Living Science Institute [18]. The intakes of the six food groups (grains, meat/fish/egg/legume, vegetables, fruits, milk/dairy products, and oils/fats/sugars) and total food were assessed. 

### 2.4. Covariates

Age, sex, family type (living alone, with spouse, with offspring, with spouse/offspring, or other), marital status (married, bereaved, or other), education (illiterate, able to read and write, elementary school, middle school, high school, college, or university and over), income level (5,000,000 won and over, 3,000,000–5,000,000 won, 2,000,000–3,000,000 won, 1,000,000–2,000,000 won, under 1,000,000 won, or unknown), smoking status (nonsmoker, ex-smoker, or current smoker), drinking status (none, 1–4 times/month, or 2–4 times/week) were investigated as general sociodemographic characteristics, and anthropometric data, including height, weight, and body mass index (BMI, kg/m^2^) were also assessed. 

### 2.5. Statistical Analysis

Differences in the general and socioeconomic data between the nonsarcopenic and sarcopenic groups were compared using Student’s *t*-test for continuous variables and the chi-squared test for categorical variables. Comparisons of the least squares mean (LSmeans) of food and nutrient intakes between the two groups were performed using a general linear model (GLM) adjusting for age, sex, and weight. The differences of nutrient intake percentages according to the 2020 Dietary Reference Intakes for Koreans (KDRIs) [19] between the two groups were compared using Student’s *t*-test. Among the KDRIs, estimated average requirement (EAR) for energy, adequate intake (AI) for fiber and sodium, and recommended nutrient intake (RNI) for other nutrients were used. Logistic regression analysis was applied to determine the risk of sarcopenia according to each food group intake and nutrient intake by evenly dividing the participants into quartiles. Multivariable analysis was performed using two models: model 1 adjusted for age and sex; and model 2 adjusted for age, sex, BMI, family type, marital status, education level, income level, smoking status, and drinking status. All statistical analyses were performed using SAS (version 9.4, SAS Institute, Cary, NC, USA), and the significance level for the analyses was set at *p* < 0.05.

## 3. Results

The characteristics of the nonsarcopenic and sarcopenic groups are presented in Table 1. The prevalence of sarcopenia in all subjects was 13.9% (*n* = 111), and there was no significant difference by gender. The mean age was significantly higher in the sarcopenic group than in the nonsarcopenic group (*p* < 0.0001), and the prevalence of sarcopenia increased with age. Body weight, BMI, muscle mass, and handgrip strength were significantly lower in the sarcopenic group than in the nonsarcopenic group. 

The comparison of food group intakes between the nonsarcopenic and sarcopenic groups are presented in Table 2. The intakes of vegetables and total food were significantly lower in the sarcopenic group than in the nonsarcopenic group (*p* < 0.05). However, the intakes of grains, meats/fish/eggs/legumes, fruits, milk/dairy products, and oils/fats/sugars did not show any difference between the sarcopenic and nonsarcopenic groups. 

The comparison of nutrient intakes between the two groups after being adjusted for age, sex, and weight are shown in Table 3. Energy intake was significantly lower in the sarcopenic group than in the nonsarcopenic group (*p* = 0.0152), and carbohydrates intake showed significant differences between the two groups (*p* < 0.05). 

The intake percentages of each nutrient according to the KDRIs between the two groups are shown in Table 4. The intake percentages of energy and protein compared to KDRIs levels were significantly lower in the sarcopenic group than in the nonsarcopenic group (79.1% vs. 87.5%, *p* = 0.0031 for energy; 93.0% vs. 103.5%, *p* = 0.0038 for protein). The intake percentages of carbohydrate, fiber, zinc, vitamin A, vitamin B_2_, and folate of the sarcopenic group were significantly lower than the nonsarcopenic group (*p* < 0.05).

Table 5 shows adjusted odds ratio (OR) and 95% confidence interval (CI) for the associations between sarcopenia risk and food group quartile intake. The risk of sarcopenia was lower in the highest quartile for the intake of meat/fish/egg/legume compared to the lowest quartile of that food group in the multivariable adjusted model (OR = 0.50, 95% CI: 0.26–0.97, *p* for trend = 0.0475). When compared with the lowest quartile of vegetable and total food intakes, there was an inverse association with sarcopenia in the highest quartile group (OR = 0.28, 95% CI: 0.13–0.59, *p* for trend = 0.0006 for vegetables; OR = 0.31, 95% CI: 0.15–0.65, *p* for trend = 0.0038 for total food intake).

Table 6 shows that higher intakes of energy, carbohydrates, proteins, fiber, zinc, carotene, and vitamin B_6_ are associated with a lower risk of sarcopena. The highest quartile of energy had a 0.44-fold lower prevalence of sarcopenia compared to the lowest quartile in the multivariate adjusted model (95% CI: 0.22–0.90, *p* for trend = 0.0089). In the cases of carbohydrate, protein, and fiber intakes, the highest quartile intake was associated with a lower prevalence of sarcopenia compared to the lowest quartile (OR = 0.40, 95% CI: 0.21–0.77, *p* for trend = 0.0048 for carbohydrate; OR = 0.44, 95% CI: 0.20–0.93, *p* for trend = 0.0222 for protein; and OR = 0.48, 95% CI: 0.25–0.93, *p* for trend = 0.0421 for fiber).

Participants in the highest quartile of zinc intake had a 0.39-fold lower risk of sarcopenia than participants in the lowest quartile of zinc intake (95% CI: 0.19–0.80, *p* for trend = 0.0074). The highest quartile of carotene and vitamin B_6_ intake was 0.49-fold and 0.45-fold lower for the risk of sarcopenia compared to the lowest quartile, respectively (95% CI: 0.26–0.93, *p* for trend = 0.0397 for carotene, 95% CI: 0.22–0.91, *p* for trend = 0.0247 for vitamin B_6_). 

## 4. Discussion

In this study, we found that a high intake of the meat/fish/eggs/legumes and vegetable food groups, total food intake, and several nutrients (energy, carbohydrate, protein, fiber, zinc, carotene, and vitamin B_6_) was associated with the lower prevalence of sarcopenia in the Korean elderly.

Among the food groups, meat/fish/eggs/legumes, vegetables, and fruits are very important for body composition, especially for muscle mass and metabolism [20]. As for the consumption of meat, a significant difference between the Korean population and Western populations should be considered. The traditional Korean dietary pattern is characterized by a low meat and high rice consumption unlike Western dietary pattern [21]. Therefore, it is recommended that the elderly in Korea consume meat that provides high-quality protein to maintain muscle mass and prevent chronic diseases. A previous cross-sectional study using the Korea National Health and Nutrition Examination Survey data showed an adverse associations between vegetable and fruit intake and sarcopenia [22]. A prospective cohort study in community-dwelling older people in Hong Kong also reported results that are in accordance with those of the present study, presenting inverse associations between vegetables and fruits and the prevalence of sarcopenia [23]. Furthermore, the meat, fish, and vegetable dietary pattern was positively associated with pre-frailty and exhaustion in the Korean elderly population [17]. Consumption of fruits and vegetables has a positive effect on sarcopenia by preventing inflammation and acidosis [24], and the intake of phytochemicals rich in fruits and vegetables increased grip strength and physical performance [12].

Among the nutrients, energy, carbohydrate, protein, fiber, zinc, carotene, and vitamin B_6_ showed adverse associations with the prevalence of sarcopenia. High energy is known to have a beneficial effect on the risk of sarcopenia [25]. This may be because energy deficiency compromises mitochondrial energy metabolism, which results in muscle fatigue, muscle weakness, and muscle atrophy [26] Another study found that higher energy intake and higher physical activity were independently associated with a reduced risk of sarcopenia in the elderly than in the younger group [13]. A recent systematic review reported that older adults with sarcopenia had significantly lower energy and carbohydrate consumptions than those without sarcopenia [27]. An intervention study suggested a preventive effect of carbohydrate and protein supplementation on muscle protein loss in bedridden patients [28]. In general, preventive guidelines for sarcopenia recommend an increased need for dietary protein because protein intake can stimulate muscle synthesis. In particular, the elderly needs more protein to prevent sarcopenia because their metabolic efficiency is low [29]. A positive association between dietary protein intake and muscle mass has been consistently reported, regardless of study designs and populations [30]. However, despite the emphasis on dietary protein intake for the prevention of sarcopenia, the status of dietary protein consumption in the Korean elderly has not met the recommended daily allowance (RDA). According to Park (2018), 47.9% of men and 60.1% of women had an insufficient protein intake based on the RDA [31]. Therefore, various methods to increase the protein intake in the Korean elderly should be explored. Fiber is abundant in fruits and vegetables, and the risk of sarcopenia has been reduced in older Chinese adults with high ”vegetable-fruit” dietary pattern scores [23]. An inverse association between the consumption of fruits and sarcopenia has also been observed in elderly people living in low-and middle-income countries [32]. The Women’s Health and Aging Study reported that carotenoid and α-tocopherol levels were associated with muscle strength in older women [33]. These results suggest that oxidative stress is a major mechanism of sarcopenia, therefore an intake of antioxidants such as carotenoids and vitamin C may prevent skeletal muscle damage [34]. Our study also demonstrated an adverse association between zinc intake and sarcopenia. Zinc deficiency contributes to the pathogenesis of anorexia nervosa [35]. Zinc has been found to stimulate appetite and may play a role in the prevention against degenerative diseases, such as sarcopenia and cachexia [35]. A narrative review elucidated the therapeutic effect of the oral zinc administration on taste disorders. Carbonic anhydrase IV, a zinc metalloenzyme, has been reported to play a role in ion transport, saliva production and secretion, and saliva pH regulation [36]. Taken together, the deficiency in dietary zinc from an inadequate diet can lead to the loss of appetite, leading to a vicious cycle of malnutrition among the elderly. The Maastricht Sarcopenia Study reported that individuals with sarcopenia had lower vitamin B_6_ intake and higher homocysteine levels than those without sarcopenia [37]. Vitamin B_6_, vitamin B_12_, and folate are known cofactors in homocysteine metabolism [37], and a recent review article elucidated that folate deficiency can contribute to the development of homocysteinemia [38]. A previous study using the Longitudinal Aging Study Amsterdam data showed an association between increased homocysteine levels and reduction in grip strength in men [39]. In addition, it has been hypothesized that higher homocysteine levels may aggravate muscle protein degradation and physical functioning in the elderly [40].

The strengths of the present study are as follows: (1) it uses nationwide data of the community-dwelling Korean elderly, and (2) it uses accurate muscle mass measurements using DEXA. However, since DEXA is exposed to radiation, an alternative method to measure muscle mass is needed. If bioelectrical impedance analysis (BIA) is used, more subjects can be involved. However, there are currently no national data linking BIA body composition with 24-h recall or dietary record. Additionally, this study has several limitations: (1) the cross-sectional study design leads to the uncertainty of a causal relationship; therefore, future prospective studies are needed to confirm the causal relationship between sarcopenia and dietary factors in the Korean elderly population, (2) it is difficult to assess exact energy intake using the 24-h recall method, and (3) the nutritional survey was a subcohort of KFACS, a random sampling cohort, and 48% of the total subjects were excluded in our analysis. It may not be representative of the Korean elderly. However, characteristics of the included subjects (52%) and excluded subjects (48%) are very similar in gender, age, height, weight, and other social factors.

According to the results of this study, sufficient consumption of nutrients through various protein source foods and vegetables will help prevent sarcopenia in the Korean elderly. This study can provide basic data for establishing dietary guidelines for the prevention of sarcopenia in the Korean elderly. 

## 5. Conclusions

The present study was conducted to assess of the effects of dietary factors on sarcopenia among the Korean elderly using nationwide data. A high intake of protein-source food, vegetables, total food intake, energy, carbohydrates, proteins, fiber, zinc, carotene, and vitamin B_6_ were associated with lower prevalence of sarcopenia in the Korean elderly.

## Figures and Tables

**Table 1:** The characteristics of the participants.

Variables	Nonsarcopenic			Sarcopenic			*p*-Value
	(*n* = 690)			(*n* = 111)			
Sex, *n* (%)							0.2477
Men	326	(47.2)		59	(53.2)		
Women	364	(52.8)		52	(46.8)		
Age (year) ^(^^1)^	76.0	±	0.2	78.2	±	0.4	<0.0001
Age group, *n* (%)							<0.0001
70–74 years	282	(40.9)		20	(18.0)		
75–79 years	247	(35.8)		40	(36.0)		
80–84 years	161	(23.3)		51	(46.0)		
Education level, *n* (%)							0.151
Illiterate	27	(3.9)		7	(6.3)		
Able to read and write	125	(18.1)		11	(9.9)		
Elementary	159	(23.0)		33	(29.7)		
Middle school	116	(16.8)		14	(12.6)		
High school	124	(18.0)		25	(22.5)		
College	31	(4.5)		6	(5.4)		
University and above	108	(15.7)		15	(13.5)		
Family type, *n* (%)							0.5803
Alone	172	(24.9)		30	(27.0)		
with spouse	357	(51.7)		61	(55.0)		
with offspring	60	(8.7)		8	(7.2)		
with spouse and offspring	89	(12.9)		12	(10.8)		
other	12	(1.7)		0	(0.0)		
Marital status, *n* (%)							0.8966
Married	463	(67.1)		72	(64.9)		
bereaved	209	(30.3)		36	(32.4)		
Others	18	(2.6)		3	(2.7)		
Smoking status, *n* (%)							0.0156
None	436	(63.2)		60	(54.1)		
Ex-smoker	226	(32.8)		40	(36.0)		
Current smoker	28	(4.1)		11	(9.9)		
Drinking status, *n* (%)							0.372
None	438	(63.5)		78	(70.3)		
≥1 times/month	124	(18.0)		17	(15.3)		
2–4 times/week	128	(18.6)		16	(14.4)		
Income, *n* (%)							0.7023
5,000,000 won and over	39	(5.7)		6	(5.4)		
3,000,000–5,000,000 won	97	(14.1)		12	(10.8)		
2,000,000–3,000,000 won	80	(11.6)		11	(9.9)		
1,000,000–2,000,000 won	154	(22.3)		21	(18.9)		
under 1,000,000 won	266	(38.6)		51	(46.0)		
unknown	54	(7.8)		10	(9.0)		
Height (cm)	158.1	±	0.3	156.4	±	0.8	0.0681
Weight (kg)	61.6	±	0.4	56.0	±	0.8	<0.0001
BMI (kg/m^2^)	24.6	±	0.1	22.9	±	0.3	<0.0001
Muscle mass (kg/m^2^)	6.56	±	0.04	5.65	±	0.07	<0.0001
Handgrip strength (kg)	24.9	±	0.3	19.0	±	0.4	<0.0001
Gait speed (m/s)	0.94	±	0.01	1.06	±	0.03	0.0003

^(1)^ mean ± standard error. The continuous data are presented as the mean ± standard error.

**Table 2:** Comparison of food group intakes.

Food Group (g)	Nonsarcopenic (*n* = 690)	Sarcopenic (*n* = 111)	*p*-Value *
	LSmeans ± SE	LSmeans ± SE	
Grains	862.0 ± 12.8	814.8 ± 33.1	0.1884
Meat/fish/eggs/legumes	278.9 ± 8.0	247.2 ± 20.5	0.1539
Vegetables	72.5 ± 1.6	62.6 ± 4.2	0.0304
Fruits	90.3 ± 3.9	74.3 ± 10.1	0.1432
Milk/dairy products	35.3 ± 2.4	31.4 ± 6.2	0.5629
Oils/fats/sugars	73.1 ± 2.6	70.2 ± 6.7	0.6905
Total food intake	1037.8 ± 14.1	953.7 ± 36.3	0.0329

* Comparison of values adjusted for age, sex, and weight between the two groups; LSmeans: the least squares mean; SE: standard error.

**Table 3:** Comparison of nutrient intakes.

Nutrient	Nonsarcopenic (*n* = 690)	Sarcopenic (*n* = 111)	*p*-Value *
	LSmeans ± SE	LSmeans ± SE	
Energy (kcal)	1512.1 ± 18.3	1387.9 ± 47.1	0.0152
Carbohydrate (g)	256.1 ± 3.3	237.0 ± 8.4	0.0360
Protein (g)	56.7 ± 0.9	52.9 ± 2.2	0.1226
Fat (g)	28.4 ± 0.7	25.8 ± 1.8	0.1806
Fiber (g)	6.07 ± 0.11	5.55 ± 0.29	0.0946
Cholesterol (mg)	194.7 ± 6.5	196.5 ± 16.7	0.9232
Calcium (mg)	446.8 ± 9.6	458.1 ± 24.6	0.6737
Phosphate (mg)	958.8 ± 12.7	915.8 ± 32.6	0.2228
Iron (mg)	13.0 ± 0.3	12.7 ± 0.7	0.6576
Sodium (mg)	3937.9 ± 75.5	3566.6 ± 194.3	0.0782
Potassium (mg)	2728.7 ± 40.0	2575.0 ± 103.0	0.1687
Zinc (mg)	7.22 ± 0.14	6.47 ± 0.37	0.0583
Vitamin A (R.E.)	676.2 ± 20.3	576.9 ± 52.3	0.0803
Retinol (µg)	110.6 ± 22.5	84.6 ± 57.8	0.6782
Carotenes (µg)	3609.4 ± 117.7	3107.6 ± 303.0	0.1269
Vitamin D (µg)	5.51 ± 0.30	6.06 ± 0.78	0.5181
Vitamin E (mg)	7.77 ± 0.15	7.37 ± 0.40	0.3564
Vitamin K (µg)	95.5 ± 4.9	75.9 ± 12.7	0.1570
Vitamin B_1_ (mg)	1.55 ± 0.34	1.04 ± 0.88	0.5951
Vitamin B_2_ (mg)	1.00 ± 0.02	0.91 ± 0.06	0.1843
Niacin (mg)	14.8 ± 0.2	14.2 ± 0.6	0.3311
Vitamin C (mg)	99.3 ± 2.7	105.5 ± 6.9	0.4023
Vitamin B_6_ (mg)	1.43 ± 0.03	1.37 ± 0.07	0.4341
Vitamin B_12_ (µg)	5.62 ± 0.26	5.48 ± 0.67	0.8522
Folate (µg)	296.9 ± 6.1	273.5 ± 15.7	0.1701

* Comparison of values adjusted for age, sex, and weight between the two groups; LSmeans: the least squares mean; SE: standard error.

**Table 4:** Comparison of nutrient intake percentages of the 2020 Dietary Reference Intakes for Koreans (KDRIs) ^(1)^.

Nutrient (%)	Nonsarcopenic (*n* = 690)	Sarcopenic (*n* = 111)	*p*-Value
	Mean ± SE	Mean ± SE	
Energy	87.5 ± 1.1	79.1 ± 2.6	0.0031
Protein	103.5 ± 1.7	93.0 ± 3.2	0.0038
Carbohydrate	197.3 ± 2.6	180.8 ± 6.7	0.0176
Fiber	27.3 ± 0.5	24.1 ± 1.2	0.0179
Calcium	60.2 ± 1.3	60.8 ± 3.5	0.8570
Phosphate	137.4 ± 1.9	127.9 ± 4.2	0.0640
Sodium	335.6 ± 6.8	313.8 ± 14.6	0.2239
Iron	153.9 ± 3.1	145.4 ± 9.4	0.3894
Zinc	91.1 ± 1.9	80.0 ± 3.2	0.0030
Vitamin A	105.1 ± 3.2	86.2 ± 7.1	0.0274
Vitamin B_1_	153.6 ± 33.3	94.8 ± 4.1	0.0804
Vitamin B_2_	84.6 ± 1.9	75.4 ± 3.6	0.0244
Niacin	114.8 ± 2.0	108.5 ± 4.4	0.2288
Vitamin C	99.8 ± 2.6	102.5 ± 7.9	0.7425
Vitamin B_6_	99.1 ± 1.8	91.0 ± 4.5	0.1008
Vitamin B_12_	234.9 ± 11.1	223.3 ± 22.0	0.6379
Folate	74.7 ± 1.6	65.6 ± 3.2	0.0111

^(1)^ Energy, estimated average requirement (EAR); fiber and sodium, adequate intake (AI); other nutrients, recommended nutrient intake (RNI) used as a dietary Reference Intakes for Koreans; SE: standard error; *p*-value: calculated using Student’s *t*-test.

**Table 5:** Odds ratios (OR) and 95% confidence intervals (CI) of sarcopenia by quartiles (Q) of food group intake and total food intake.

Food Group		Median (g)	Total (*n*)	Sarcopenia (*n*)	Age, Sex-Adjusted OR (95% CI)		Multi-Adjusted OR (95% CI)	
Grains	Q1	489	200	35	1.00		1.00	
	Q2	719	200	26	0.73	(0.42–1.29)	0.66	(0.36–1.22)
	Q3	921	201	23	0.65	(0.36–1.17)	0.56	(0.29–1.08)
	Q4	1224	200	27	0.74	(0.42–1.32)	0.57	(0.31–1.07)
	*p* for trend				0.3128		0.089	
Meat/fish /eggs/legumes	Q1	68	200	36	1.00		1.00	
	Q2	171	200	29	0.74	(0.43–1.28)	0.62	(0.34–1.13)
	Q3	293	201	24	0.61	(0.34–1.09)	0.47	(0.25–0.89)
	Q4	505	200	22	0.56	(0.30–1.02)	0.50	(0.26–0.97)
	*p* for trend				0.0579		0.0475	
Vegetables	Q1	27	200	37	1.00		1.00	
	Q2	53	200	32	0.85	(0.50–1.46)	0.85	(0.48–1.51)
	Q3	78	200	28	0.76	(0.44–1.33)	0.66	(0.36–1.22)
	Q4	118	201	14	0.34	(0.17–0.66)	0.28	(0.13–0.59)
	*p* for trend				0.0014		0.0006	
Fruits	Q1	0	213	35	1.00		1.00	
	Q2	42	188	30	0.96	(0.56–1.65)	0.98	(0.54–1.77)
	Q3	88	200	24	0.68	(0.39–1.21)	0.66	(0.35–1.26)
	Q4	192	200	22	0.68	(0.38–1.22)	0.72	(0.38–1.35)
	*p* for trend				0.1408		0.2253	
Milk/dairy products	Q1	0	525	80	1.00		1.00	
	Q2	20	76	9	0.72	(0.34–1.53)	0.81	(0.36–1.80)
	Q3	120	200	22	0.71	(0.43–1.18)	0.70	(0.40–1.22)
	*p* for trend				0.2003		0.2189	
Oils/fats/ sugars	Q1	5	200	33	1.00		1.00	
	Q2	38	200	30	0.86	(0.50–1.49)	0.73	(0.40–1.33)
	Q3	75	201	26	0.78	(0.44–1.37)	0.78	(0.42–1.46)
	Q4	148	200	22	0.69	(0.38–1.24)	0.57	(0.29–1.10)
	*p* for trend				0.2071		0.1284	
Total food intake	Q1	617	200	35	1.00		1.00	
	Q2	887	200	31	0.90	(0.52–1.55)	0.82	(0.45–1.50)
	Q3	1101	201	31	0.90	(0.52–1.57)	0.92	(0.49–1.71)
	Q4	1437	200	14	0.36	(0.18–0.72)	0.31	(0.15–0.65)
	*p* for trend				0.0059		0.0038	

Multi-adjusted: adjusted for age, sex, BMI, family type, marital status, education level, income level, smoking status, and drinking status.

**Table 6:** Odds ratios (OR) and 95% confidence intervals (CI) of sarcopenia by quartiles (Q) of nutrient intake.

Nutrient		Median	Total	Sarcopenia	Age, Sex-Adjusted		Multi-Adjusted	
			(*n*)	(*n*)	OR (95% CI)		OR (95% CI)	
Energy	Q1	928.1	200	32	1.00		1.00	
	Q2	1301.3	200	36	1.24	(0.72–2.13)	1.13	(0.63–2.02)
	Q3	1590.5	201	25	0.79	(0.43–1.43)	0.61	(0.32–1.17)
	Q4	2062.3	200	18	0.55	(0.28–1.06)	0.44	(0.22–0.90)
		*p* for trend			0.0379		0.0089	
Carbohydrate	Q1	160.3	200	40	1.00		1.00	
	Q2	220.4	200	26	0.63	(0.36–1.11)	0.63	(0.34–1.15)
	Q3	273.4	201	24	0.57	(0.32–1.00)	0.51	(0.27–0.94)
	Q4	349.9	200	21	0.49	(0.27–0.89)	0.40	(0.21–0.77)
		*p* for trend			0.0175		0.0048	
Protein	Q1	30.1	199	29	1.00		1.00	
	Q2	47.2	201	35	1.25	(0.72–2.19)	1.11	(0.61–2.01)
	Q3	59.8	201	31	1.10	(0.62–1.95)	0.87	(0.46–1.65)
	Q4	81.8	200	16	0.50	(0.25–1.00)	0.44	(0.20–0.93)
		*p* for trend			0.0429		0.0222	
Fat	Q1	9.6	200	29	1.00		1.00	
	Q2	19.1	199	34	1.23	(0.71–2.14)	1.18	(0.65–2.15)
	Q3	29.3	201	26	0.94	(0.52–1.70)	0.77	(0.41–1.46)
	Q4	48.2	201	22	0.76	(0.41–1.41)	0.69	(0.35–1.36)
		*p* for trend			0.2394		0.1489	
Fiber	Q1	2.9	200	36	1.00		1.00	
	Q2	4.8	200	26	0.67	(0.38–1.17)	0.65	(0.35–1.18)
	Q3	6.4	201	28	0.79	(0.45–1.38)	0.69	(0.37–1.27)
	Q4	9.3	200	21	0.53	(0.29–0.96)	0.48	(0.25–0.93)
		*p* for trend			0.0603		0.0421	
Cholesterol	Q1	32.6	200	30	1.00		1.00	
	Q2	102.0	200	33	1.09	(0.63–1.90)	1.04	(0.57–1.88)
	Q3	199.5	201	23	0.71	(0.39–1.28)	0.7	(0.37–1.34)
	Q4	407.5	200	25	0.82	(0.46–1.48)	0.78	(0.40–1.54)
		*p* for trend			0.3441		0.3526	
Calcium	Q1	208.9	200	30	1.00		1.00	
	Q2	341.6	200	23	0.76	(0.42–1.37)	0.70	(0.37–1.31)
	Q3	468.0	201	31	1.02	(0.58–1.79)	0.88	(0.48–1.64)
	Q4	706.6	200	27	0.93	(0.52–1.66)	0.89	(0.47–1.69)
		*p* for trend			0.9623		0.9513	
Phosphate	Q1	574.8	200	31	1.00		1.00	
	Q2	833.2	200	32	1.07	(0.62–1.87)	0.88	(0.48–1.61)
	Q3	1013.6	201	26	0.89	(0.50–1.61)	0.69	(0.36–1.32)
	Q4	1323.0	200	22	0.69	(0.37–1.29)	0.57	(0.28–1.14)
		*p* for trend			0.2033		0.0885	
Iron	Q1	6.4	200	29	1.00		1.00	
	Q2	10.0	201	35	1.29	(0.74–2.24)	1.47	(0.80–2.71)
	Q3	13.4	199	25	0.88	(0.48–1.60)	0.75	(0.39–1.47)
	Q4	20.0	201	22	0.79	(0.42–1.46)	0.76	(0.38–1.51)
		*p* for trend			0.246		0.1631	
Sodium	Q1	1987.2	200	34	1.00		1.00	
	Q2	3071.4	200	36	1.10	(0.64–1.87)	1.11	(0.62–1.96)
	Q3	4031.5	201	19	0.48	(0.26–0.90)	0.42	(0.21–0.83)
	Q4	6035.7	200	22	0.55	(0.30–1.01)	0.49	(0.25–0.96)
		*p* for trend			0.0157		0.0107	
Potassium	Q1	1494.7	200	35	1.00		1.00	
	Q2	2264.1	200	30	0.91	(0.52–1.57)	0.85	(0.47–1.54)
	Q3	3000.0	201	22	0.58	(0.32–1.06)	0.55	(0.28–1.05)
	Q4	3945.2	200	24	0.69	(0.38–1.27)	0.63	(0.32–1.24)
		*p* for trend			0.1264		0.1083	
Zinc	Q1	3.9	199	33	1.00		1.00	
	Q2	5.7	201	30	0.93	(0.53–1.61)	0.92	(0.51–1.67)
	Q3	7.3	201	29	0.85	(0.48–1.50)	0.78	(0.42–1.44)
	Q4	10.2	200	19	0.53	(0.28–1.00)	0.39	(0.19–0.80)
		*p* for trend			0.0451		0.0074	
Vitamin A	Q1	174.9	200	34	1.00		1.00	
	Q2	398.0	201	36	1.14	(0.67–1.92)	0.92	(0.52–1.64)
	Q3	677.0	200	19	0.57	(0.31–1.05)	0.44	(0.23–0.86)
	Q4	1239.2	200	22	0.69	(0.38–1.24)	0.54	(0.28–1.03)
		*p* for trend			0.0835		0.0298	
Retinol	Q1	4.0	199	36	1.00		1.00	
	Q2	19.6	201	28	0.71	(0.41–1.23)	0.71	(0.39–1.30)
	Q3	58.0	201	19	0.45	(0.25–0.83)	0.40	(0.21–0.77)
	Q4	146.0	200	28	0.72	(0.41–1.25)	0.67	(0.36–1.26)
		*p* for trend			0.4057		0.3352	
Carotene	Q1	844.3	200	38	1.00		1.00	
	Q2	1973.3	200	29	0.74	(0.43–1.27)	0.64	(0.36–1.15)
	Q3	3539.6	201	22	0.58	(0.33–1.04)	0.49	(0.26–0.91)
	Q4	6856.5	200	22	0.59	(0.33–1.06)	0.49	(0.26–0.93)
		*p* for trend			0.0874		0.0397	
Vitamin D	Q1	0	202	31	1.00		1.00	
	Q2	1.6	199	22	0.70	(0.38–1.26)	0.56	(0.29–1.06)
	Q3	4.9	203	27	0.92	(0.52–1.63)	0.82	(0.44–1.53)
	Q4	12.3	197	31	1.01	(0.58–1.76)	0.92	(0.51–1.68)
		*p* for trend			0.5653		0.6279	
Vitamin E	Q1	3.4	207	33	1.00		1.00	
	Q2	5.9	192	33	1.08	(0.63–1.86)	1.02	(0.56–1.85)
	Q3	8.3	202	26	0.84	(0.47–1.49)	0.71	(0.38–1.33)
	Q4	12.2	200	19	0.61	(0.33–1.15)	0.53	(0.26–1.06)
		*p* for trend			0.0873		0.0438	
Vitamin K	Q1	9.0	200	36	1.00		1.00	
	Q2	37.5	200	26	0.75	(0.43–1.32)	0.70	(0.38–1.26)
	Q3	76.0	201	29	0.83	(0.48–1.44)	0.75	(0.41–1.38)
	Q4	185.0	200	20	0.55	(0.30–1.00)	0.51	(0.27–0.98)
		*p* for trend			0.0678		0.0707	
Vitamin B_1_	Q1	0.5	201	27	1.00		1.00	
	Q2	0.8	199	37	1.54	(0.88–2.69)	1.52	(0.84–2.76)
	Q3	1.0	201	19	0.76	(0.40–1.45)	0.62	(0.31–1.25)
	Q4	1.5	200	28	1.06	(0.58–1.94)	1.12	(0.58–2.15)
		*p* for trend			0.6475		0.7303	
Vitamin B_2_	Q1	0.5	195	32	1.00		1.00	
	Q2	0.8	202	34	1.03	(0.60–1.77)	1.05	(0.58–1.89)
	Q3	1.0	204	21	0.64	(0.35–1.17)	0.55	(0.28–1.07)
	Q4	1.5	200	24	0.71	(0.39–1.30)	0.67	(0.34–1.32)
		*p* for trend			0.1398		0.1103	
Niacin	Q1	7.4	198	30	1.00		1.00	
	Q2	11.5	200	29	0.99	(0.56–1.76)	0.93	(0.50–1.73)
	Q3	15.9	202	29	0.94	(0.53–1.68)	0.77	(0.41–1.45)
	Q4	22.3	201	23	0.76	(0.40–1.45)	0.66	(0.33–1.33)
		*p* for trend			0.384		0.202	
Vitamin C	Q1	34.8	199	26	1.00		1.00	
	Q2	67.4	201	35	1.43	(0.82–2.51)	1.57	(0.84–2.92)
	Q3	106.1	201	23	0.90	(0.49–1.65)	0.86	(0.44–1.66)
	Q4	173.9	200	27	1.07	(0.59–1.94)	1.07	(0.56–2.07)
		*p* for trend			0.768		0.6419	
Vitamin B_6_	Q1	0.7	202	34	1.00		1.00	
	Q2	1.1	196	30	0.91	(0.52–1.58)	0.90	(0.49–1.66)
	Q3	1.5	203	29	0.87	(0.49–1.53)	0.85	(0.45–1.60)
	Q4	2.1	200	18	0.51	(0.27–0.97)	0.45	(0.22–0.91)
		*p* for trend			0.0431		0.0247	
Vitamin B_12_	Q1	0.6	199	25	1.00		1.00	
	Q2	2.3	201	29	1.23	(0.69–2.21)	1.02	(0.55–1.91)
	Q3	4.9	201	28	1.22	(0.68–2.21)	1.00	(0.53–1.89)
	Q4	11.7	200	29	1.24	(0.69–2.24)	1.17	(0.62–2.21)
		*p* for trend			0.6002		0.5874	
Folate	Q1	144.1	199	33	1.00		1.00	
	Q2	223.8	201	23	0.70	(0.39–1.26)	0.55	(0.29–1.06)
	Q3	304.4	200	36	1.13	(0.66–1.96)	1.01	(0.56–1.84)
	Q4	451.6	201	19	0.56	(0.30–1.04)	0.51	(0.26–1.01)
		*p* for trend			0.1518		0.1789	

Multi-adjusted: adjusted for age, sex, BMI, family type, marital status, education level, income level, smoking status, and drinking status.

## Data Availability

All datasets used and/or analyzed during the current study are available from the corresponding author on reasonable request.
